# Healthcare Utilization and Knee Osteoarthritis Symptoms among Urban Older Malaysian

**DOI:** 10.3390/ijerph18073777

**Published:** 2021-04-04

**Authors:** Siti Salwana Kamsan, Devinder Kaur Ajit Singh, Maw Pin Tan, Saravana Kumar

**Affiliations:** 1 Physiotherapy Program & Centre for Healthy Ageing & Wellness, Faculty of Health Sciences, Universiti Kebangsaan Malaysia, Kuala Lumpur 50300, Malaysia; sitisalwana@iium.edu.my; 2 Department of Physical Rehabilitation Sciences, Faculty of Allied Health Sciences, International Islamic University Malaysia, Pahang 25200, Malaysia; 3 Division of Geriatric Medicine, Department of Medicine, Faculty of Medicine, University of Malaya, Kuala Lumpur 50603, Malaysia; mptan@ummc.edu.my; 4 Department of Medical Sciences, Faculty of Healthcare and Medical Sciences, Sunway University, Bandar Sunway 47500, Malaysia; 5 Allied Health and Human Performance, City East Campus, University of South Australia, Adelaide 5000, Australia; Saravana.Kumar@unisa.edu.au

**Keywords:** healthcare services, older persons, knee osteoarthritis

## Abstract

Knee osteoarthritis (OA) is a prevalent chronic disorder in the older population. While timely management is important to minimize the consequences of knee OA, information on the utilization of healthcare services among this population remains limited. Therefore, the objectives of this study were to determine the healthcare utilization and its associated factors in older persons with knee OA. Cross-sectional data from 1073 participants aged 60 years and above from the Malaysian Elders Longitudinal Research (MELoR) study were included. The utilization rate of healthcare services was quantified. Factors related to the utilization of healthcare services were determined using logistic regression analysis. Healthcare utilization among participants with knee OA was significantly higher than those without knee OA (*p* < 0.01). Outpatient usage was higher (*p* < 0.01) in comparison to inpatient and pharmacotherapy. Being married and having an income were significantly associated with seeking outpatient care (OR: 11.136, 95% CI: 1.73–52.82, *p* < 0.01) and pharmacotherapy (OR: 10.439, 95% CI: 1.187–91.812, *p* < 0.05), while females were less likely to utilize inpatient care services (OR: 0.126, 95% CI: 0.021–0.746, *p* < 0.05). The higher rate of healthcare utilization among older persons with knee OA indicates the increased healthcare needs of this population, who are commonly assumed to suffer from a benign disease.

## 1. Introduction

Knee osteoarthritis (OA) is a prevalent chronic disorder that increases in prevalence from late middle-age to old age. Its incidence is reported to vary across different geographical locations. In particular, the prevalence of knee OA among the older population in the United States, Europe, Middle East, and Asian countries is around 13% to 20% [1], 9–17% [2], 22% to 25% [3], and 10% to 38% [4,5], respectively. As age and obesity are the main risk factors of OA, these figures are projected to escalate with the global population aging and the obesity epidemic [6,7].

Knee OA was ranked as the 38th highest of 291 diseases in terms of overall burden measured by disability-adjusted life years [8]. Pain, mobility impairment, and physical inactivity are among the major consequences of knee OA that lead to functional limitation and dependency [9,10,11,12,13]. The symptoms burden associated with this chronic and disabling condition is expected to lead to increased healthcare utilization. Individuals with OA are expected to access healthcare services through both outpatient and inpatient hospital care in terms of consultations with physicians, geriatricians, orthopedic surgeons, and rehabilitation professionals [14,15].

Conservative approaches requiring multifaceted strategies including patient education, pain management, exercises, weight management, and joint protection strategies should be delivered as first-line therapy for knee OA. Typically, individuals with knee OA may use multiple treatment options such as medication, physiotherapy intervention, and exercises therapy for their symptoms management [16]. More invasive approaches involving surgical procedures are only recommended for an advanced stage of knee OA, once non-invasive treatment modalities are no longer deemed effective [17,18,19]. In addition to conventional medicine, alternative therapy has also been sought by people with knee OA as part of their management strategies [20,21].

The healthcare cost of knee OA has been consistently reported as $10,000 to $35,000 per person annually [22,23,24,25]. However, more detailed information pertaining to the utilization of healthcare resources among individuals with knee OA in developing countries remains limited. In a few studies, an increased utilization of medical care in individuals with knee OA have been reported [15,26]. In contrast, underutilization of the healthcare services in people with OA knee was reported in another study [27]. The ambiguity between studies may reflect the challenges with accessing healthcare for a condition that limits mobility and is traditionally deemed by healthcare professionals as mild and non-life threatening. As a consequence, there is an unequal and inefficient allocation of healthcare resources delivered for this condition [28].

Specifically in Malaysia, despite the observed increase in prevalence of knee OA over the past decades [29,30], the rate of healthcare usage among this population has never been explored. Quantification of healthcare utilization for individuals with knee OA, particularly among those who are approaching retirement or who have retired is vital to inform resources allocation as well as for healthcare planning and policy development. It has also been increasingly recognized that OA of major joints such as the knee is not necessarily a benign disease but instead may lead to increased risk of adverse health outcomes such as falls in older adults [31]. Therefore, the objectives of our study were to determine the healthcare utilization and its associated factors among older persons with knee OA.

## 2. Materials and Methods

### 2.1. Study Design and Data Source

This study was a retrospective study. The cross-sectional dataset from the Malaysian Elders Longitudinal Research (MELoR) study was employed in this study. Details on participant recruitment have been reported in previous studies [32,33,34,35]. Briefly, a total of 1616 participants aged 55 years and over were recruited from the 13th general election parliamentary constituencies of Pantai Valley, Petaling Jaya North, and Petaling Jaya South through simple random sampling stratified by age deciles and the three main ethnicities of Malay, Chinese, and Indian. Sociodemographic, medical history, psychological status, quality of life, and health utilization data were collected during a home-based computer assisted interview at recruitment, which occurred between 2013 and 2015.

### 2.2. Cases Selection

Cases were selected based on predetermined criteria that included age 60 years and above with available data on knee pain and knee OA symptom status. This age criteria for older persons was based on the Malaysian context where anyone aged 60 and over is considered to be elderly [36]. The presence of knee OA was determined by enquiring participants using the following question, “In the past 12 months, have you had pain, aching, or stiffness in either knee, on most days for at least one month?” Participants were excluded if they had serious medical illnesses including cancer, heart and kidney diseases, brain and any neurological disorders (dementia, stroke, and Parkinson disease), as these illnesses require complex healthcare needs, which are likely to confound rates of health utilization in our study participants [37,38].

### 2.3. Study Measure

Three types of healthcare services utilization were identified from the database, namely outpatient care, inpatient care, and pharmacotherapy. In this study, outpatient care was referred to as having a history of visiting public or private hospitals or clinics. Meanwhile, inpatient care referred to self-reported episodes of hospitalization. Pharmacotherapy referred to the consumption of prescribed or non-prescribed medicines. Sociodemographic and clinical characteristics of the participants were examined as associated factors of healthcare utilization.

### 2.4. Analysis

All analyses were performed using statistical package for social sciences (SPSS) version 23. The proportion of older persons with knee OA who had a history of using healthcare services over the past one year was determined. A comparison of healthcare usage among older persons with and without knee OA was conducted. To identify factors that were associated with healthcare utilization in this population, a multilevel binary logistic regression analysis was performed. The variables were categorized into three groups based on Andersen’s framework [39]. Age, gender, ethnicity, marital status, and educational level were categorized into predisposing factors. Income was categorized as an enabling factor, while pain severity and comorbid condition were categorized as needs factors. To perform this analysis, predisposing, enabling, and needs factors were classified into blocks 1, 2, and 3, respectively. A two tailed *p*-value less than 0.05 was interpreted as statistically significant.

## 3. Results

### 3.1. Sample Characteristics

The characteristics of the participants of this study are summarized in Table 1. A total of 1616 participants participated in the MELoR study, of which 1073 participants were aged 60 years and above and had no serious medical illnesses. Out of these, 266 participants were identified as older persons with knee OA. Based on the findings, knee OA was more prevalent among women than men (30% vs. 18%, *p* < 0.001). In addition, knee OA was less likely associated with Chinese ethnicity, higher educational levels, and absence of comorbid conditions (*p* < 0.001, *p* < 0.001 and *p* < 0.05, respectively). More than half of the older persons with knee OA reported moderate to severe pain severity (63.5%).

### 3.2. Prevalence and Associated Factors of Healthcare Utilization in Older Persons with Knee OA

The prevalence of healthcare utilization was first stratified into two groups: knee OA and non-knee OA (Table 2). The results showed that 95% of older persons with knee OA had utilized healthcare services, and the use of healthcare services by this population was high compared to older persons without knee OA (*p* < 0.01). In terms of the types of services used, a significant difference was found for outpatient care, indicating that this service was utilized more frequently in the knee OA group (*p* ≤ 0.001).

Multilevel binary logistic regression analysis demonstrated that being married and having an income were associated with the usage of outpatient services and pharmacotherapy, while being female was less likely to be associated with inpatient care services (Table 3). Based on these findings, married older persons tended to be 13.6% more likely to visit outpatient care facility (OR: 11.136, 95% CI: 1.73–52.82, *p* < 0.01). Those who had at least one source of income were found to be 43.9% more likely to use pharmacotherapy (OR: 10.439, 95% CI: 1.187–91.812, *p* < 0.05). Female older persons were 87.4% less frequently to be hospitalized as compared to males (OR: 0.126, 95% CI 0.021–0.746, *p* < 0.05).

## 4. Discussion

The objectives of this study were to determine the healthcare utilization and its associated factors among older persons with knee osteoarthritis. To our knowledge, this is the first study that examined the healthcare utilization in older persons with knee OA in Malaysia and also the first study examining the three different factors known as predisposing factor, need factor, and enabling factor that were associated with the healthcare usage in the Malaysian context.

Based on our study findings, the majority of the older persons with knee OA had utilized healthcare services in the past one year (95%), regardless of the types of services. Only a very small number of participants had not utilized the healthcare services. The reason for this minority not accessing the healthcare services is unknown. However, previous studies reported that financial issues or geographical factors could be one of the main barriers to accessing the healthcare services [40,41]. In addition, the perception that knee OA is a normal condition with aging may also have led to ignorance of seeking appropriate treatment and care [42]. As for the majority who utilize the healthcare services, seeking pain relief is the main factor for accessing the healthcare services [38].

The utilization of healthcare services was doubled in older persons with knee OA compared to those without knee OA. This finding is consistent with previous reports that showed high utilization of healthcare services among this population [14,15,26,43,44]. This finding suggests that older persons with knee OA require assistance in managing their condition. This could probably be to minimize the consequences of knee OA including pain, swelling, and stiffness of the knee, which could affect the mobility and activities of daily living [10,16].

Among the three types of healthcare services, outpatient care services had the highest rate of utilization, followed by pharmacotherapy and inpatient care. Nevertheless, only outpatient care services had the statistically significant difference, indicating that older persons with knee OA had higher utilization of this service as compared to those without knee OA. This finding is supported by previous studies [26,45]. This finding could be explained by the fact that most of the knee OA symptoms are successfully managed using conservative treatments, and people with knee OA are frequently referred to outpatient care facilities for comprehensive management and care [46,47,48]. In addition, people with knee OA are frequently referred to outpatient care facilities for continuous management and care [46,47,48].

As in other countries, outpatient care has been widely offered at primary healthcare centers by general practitioners in Malaysia. This service is normally easily accessible and in the vicinity where people live. This service can be accessed with trifling cost. The nominal fee of RM1–RM5 is charged for outpatient services in the public health sector, which makes this service extremely affordable for the local population. Moreover, this nominal fee does not apply to government employees and government pensioners, as the fee is subsidized by the government [49]. This nominal charge could be one of the factors that contribute to the high rate of outpatient services utilization in persons with knee OA in Malaysia.

Pertaining to the associated factors of healthcare utilization, our study analysis revealed that there were several factors related to the usage of outpatient care, inpatient care, and pharmacotherapy. Marital status in which being married was found to be associated with outpatient services utilization. This result suggests that older people with spouses are likely to visit outpatient care amenities as compared to those who are alone. Regardless of their health condition, being married was found to be a significant factor for healthcare utilization [50,51,52]. It can be deduced that married persons could be influenced or encouraged by their spouses or children to seek treatment for their condition and hence utilizing the available healthcare facilities [50].

As for inpatient care, female older persons with knee OA were less likely to have hospital admissions as compared to their male counterparts. This is probably due to the fact that women are more concerned about their health, which led to more positive health-seeking behaviors [53]. As a result, they tend to obtain more health advice from a variety of sources in order to maintain their well-being and to avoid unnecessary hospitalization [54]. Congruent with the results from other studies, women are found to have more physician consultations as compared to men [55,56,57,58,59].

Older persons with knee OA who had at least one source of income in our study were also more likely to take medication to manage their symptoms. This is expected, as older persons with higher socio-economic status would be more likely to adhere to pharmacotherapy in managing their illnesses [15,60,61]. In Malaysia, particularly, low household income is more prevalent in rural areas [62]. It is noteworthy that participants in our study were from urban areas, and therefore, they might not have any financial strain to acquiring medications.

Our main study limitation was that it was conducted at an urban geographical location. Given that much of the Malaysian population lives outside of urban settings, this could limit its generalizability to rural areas in Malaysia. The pattern of healthcare utilization among people with knee OA in metropolitan areas of other countries may also differ from the present study findings. Disparities in the use of healthcare facilities and services by geographical regions have been reported in previous studies [63,64]. Additionally, the healthcare utilization data were obtained based on respondents’ self-reports. Participants were asked to recall the types of healthcare services used in the past year, which could lead to recall bias. Lastly, the rate of healthcare utilization, the amount of use for each type of healthcare service, and information regarding lifestyle and related genetic factors were not obtained, as we relied on the available data that were retrospective in nature. Further studies are required to determine the volume of healthcare usage and the amount of use for each type of treatment in outpatient facilities in this population. This information could have assisted in further understanding and adding to the literature regarding the pattern and trend of healthcare utilization in older persons with knee OA.

## 5. Conclusions

In conclusion, higher healthcare usage was demonstrated among older persons with knee OA compared to those without knee OA. Outpatient care services were significantly more frequently used in comparison to inpatient care and pharmacotherapy in older persons with knee OA. Being married, having an income, and being males were found to be significantly associated with healthcare utilization for the management of knee OA. Our study findings provide information regarding the pattern of healthcare utilization and health needs in older persons with knee OA. This information is important for healthcare management teams for efficient healthcare delivery, resources, and policy planning. There may also be a need to promote evidence-based practice among healthcare providers and to provide self-management education to older persons with knee OA.

## Figures and Tables

**Table 1:** Demographic characteristics of sample.

Characteristics	Total Sample	Knee OA Group	Non-Knee OA Group	*p*-Value
	*n*	*n* (%)	*n* (%)	
	*n* = 1073	*n* = 266 (24.8)	*n* = 807 (75.2)	
Age (±SD)	70.17 (±6.66)	69.87 (±6.67)	70.27 (±6.66)	0.355 ^#^
Age range (years)				
60–69	526	137 (26.05)	389 (73.95)	0.750 ^+^
70–79	454	108 (23.79)	346 (76.21)	
80–89	86	20 (23.26)	66 (76.74)	
90 and above	7	1 (14.29)	6 (85.71)	
Gender				
Males	453	80 (17.67)	373 (82.34)	<0.001 ^+,^**
Females	620	186 (30)	434 (70)	
Ethnic				
Malay	358	109 (30.45)	249 (69.55)	<0.001 ^+,^**
Chinese	398	71 (17.84)	327 (82.16)	
India	309	84 (27.18)	225 (72.82)	
Others	8	2 (25)	6 (75)	
Marital status				
Single	65	17 (26.15)	48 (73.85)	0.325 ^+^
Married	764	177 (23.17)	587 (76.83)	
Divorce	25	7 (28)	18 (72)	
Widowed	213	64 (30.05)	149 (69.95)	
Others	6	1 (16.67)	5 (83.33)	
Educational level				
No formal education	43	12 (27.91)	31 (72.09)	0.001 ^+,^**
Primary	259	87 (33.59)	172 (66.41)	
Secondary	439	102 (23.23)	337 (76.77)	
Post-secondary	64	16 (25)	48 (75)	
College/University	268	49 (18.28)	219 (81.72)	
Income				
≥One source of income	1019	258 (25.32)	761 (74.68)	0.081 ^+^
No source of income	54	8 (14.81)	46 (85.19)	
Comorbidities				
≥One	894	234 (26.17)	660 (73.83)	0.019 ^+,^*
None	179	32 (17.88)	147 (82.12)	
Knee pain severity				
No pain	-	8 (3)	-	-
Mild pain	-	89 (33.5)	-	-
Moderate pain	-	132 (49.6)	-	-
Severe pain	-	37 (13.9)	-	-

^#^: *p*-value for Mann–Whitney *U* test; ^+^: *p*-value for Pearson chi-square; *: *p* < 0.05; **: *p* < 0.001.

**Table 2:** Healthcare utilization in older persons with and without knee osteoarthritis.

Variables	Knee OA, *n* (%)	Non-Knee OA, *n* (%)	*p*-Value
	*n* = 266 (24.8)	*n* = 807 (75.2)	
Utilization of healthcare services			
Yes	252 (26.17)	711 (73.83)	0.002 ^+,^*
No	14 (12.73)	96 (87.27)	
Types of services used			
Outpatient visits	245 (26.95)	664 (73.05)	0.001 ^+,^**
Inpatient care	8 (24.24)	25 (75.76)	0.941 ^+^
Pharmacotherapy	170 (25.56)	495 (74.44)	0.589 ^++^

^+^: *p*-Value for Pearson chi-square; ^++^: *p*-Value for Fisher Exact Test; *: *p* ≤ 0.01; **: *p* ≤ 0.001.

**Table 3:** Associated factors of healthcare utilization in older persons with knee OA.

Variable	B	SE (b)	*p*	Exp (B)	95% CI	
					Upper	Lower
Outpatient care						
Age	0.065	0.093	0.484	1.067	0.890	1.279
Age range	−0.606	1.017	0.551	0.545	0.074	4.000
Gender (F)	0.872	0.638	0.171	2.393	0.685	8.351
Ethnic	−0.244	0.683	0.720	0.783	0.205	2.986
Marital status	2.410	0.904	0.008 *	11.136	1.893	65.517
Educational level	1.836	0.946	0.052	6.273	0.983	40.036
Income	−20.417	13,678.627	0.999	0.000	0.000	
Pain severity	0.565	0.587	0.336	1.760	0.557	5.561
Medical condition	−0.347	0.872	0.691	0.707	0.128	3.901
Inpatient care						
Age	−0.012	0.154	0.939	0.988	0.731	1.336
Age range	−0.066	1.737	0.970	0.936	0.031	28.205
Gender (F)	−2.069	0.906	0.022 *	0.126	0.021	0.746
Ethnic	0.673	1.026	0.512	1.960	0.263	14.630
Marital status	0.262	0.545	0.631	1.300	0.446	3.782
Educational level	0.84	0.339	0.805	1.087	0.559	2.113
Income	−16.161	12,833.2	0.999	0.000	0.000	
Pain severity	−0.547	0.561	0.329	0.579	0.193	1.737
Medical condition	−1.354	0.861	0.116	0.258	0.048	1.397
Pharmacotherapy						
Age	0.044	0.052	0.403	1.045	0.943	1.158
Age range	−0.527	0.591	0.373	0.591	0.186	1.880
Gender (F)	0.058	0.341	0.864	1.060	0.544	2.067
Ethnic	0.697	0.390	0.074	2.007	0.935	4.306
Marital status	−0.485	0.847	0.567	0.615	0.117	3.239
Educational level	0.134	0.716	0.852	1.143	0.281	4.651
Income	2.346	1.109	0.034 *	10.439	1.187	91.812
Pain severity	−0.394	0.321	0.219	0.674	0.360	1.264
Medical condition	0.332	0.423	0.433	1.393	0.608	3.192

*: *p* < 0.05.

## Data Availability

The data presented in this study are available on request from corresponding author. The data are not publicly available due to confidentiality concerns.
